# Excess Properties of Aqueous Dilutions of Ammonium- and Phosphonium-Based Deep Eutectic Solvents

**DOI:** 10.1021/acs.jced.6c00067

**Published:** 2026-04-28

**Authors:** Xusheng Zhou, Wenjia Zhang, Manuel Cerro Carracedo, Rafael J. Jiménez-Riobóo, M. Luisa Ferrer, María C. Gutiérrez, Francisco del Monte

**Affiliations:** 16379 Instituto de Ciencia de Materiales de Madrid-ICMM, Consejo Superior de Investigaciones Científicas-CSIC, Campus de Cantoblanco, Madrid 28049, Spain

## Abstract

This work focused first on the measurement at 25 °C and ambient pressure of the thermal conductivities (*k*), thermal diffusivities (α), and heat capacities (C_p_) of aqueous dilutions of two deep eutectic solvents (DESs). The first one composed of choline chloride (ChCl) and urea (U) in a 1:2 molar ratio (ChCl:2U), and the second one composed of methyltriphenylphosphoniumbromide (M3PhPBr) and glycerol (Gly) in a 1:3 molar ratio (M3PhPBr:3Gly). Then, we compared the deviation from ideality of the thermal conductivity (Δ*k*) with those experienced by other excess properties, including excess molar volume (*V* ^E^), excess viscosity (ln η^E^), and excess adiabatic compressibility (Δβ_S_). The aim was finding some correlation among them in the sign of the deviation and/or the specific DES content at which the maximum deviation from ideality is reached. Moreover, we have compared the hypersonic velocities (ν_H_) obtained from Brillouin spectroscopy with regular ultrasonic velocities (ν_U_) to identify the DES content for which, in terms of relaxation, the mixture moves from a purely dissipative fluid to an “elastic medium”. Finally, we also investigated whether ^1^H NMR spectroscopy could also be used to identify the DES content for which the deviation of ideality was the maximum.

## Graphical abstract

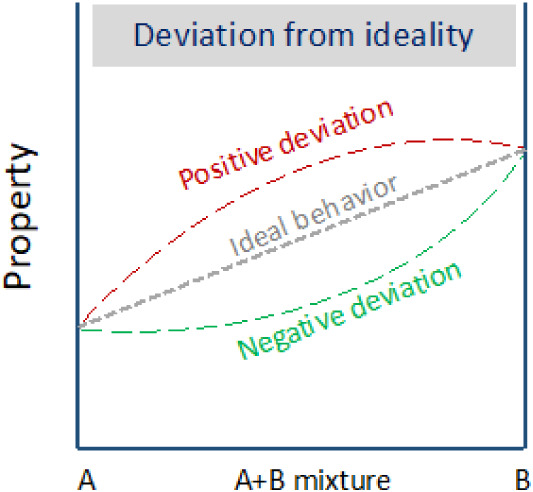

## 1 Introduction

Deep eutectic solvents (DESs)^1^ have lately attracted great attention because, for certain compositions (like the so-called natural DESs—NADESs),^2–4^ they may offer a more sustainable, cost-effective, and environmentally benign alternative to traditional organic solvents and ionic liquids, particularly interesting in the fields of biotechnology and biomedicine.^5–8^ These exceptional properties are a direct consequence of the intricate molecular architecture formed within the mixture. A DES is typically formed by simply mixing a hydrogen bond acceptor (HBA)—often a quaternary ammonium salt like choline chloride (ChCl)—with a hydrogen bond donor (HBD)—such as urea (U), carboxylic acids, amines, or polyols like glycerol (Gly).^9^ The crucial factor is the formation of strong, specific hydrogen bond (HB) interactions between the HBA and HBD molecules.^10–14^ These interactions effectively lower the lattice energy of each component, preventing the mixture from crystallizing at temperatures at which the individual pure components would solidify. Moreover, the tunability of these interactions, by simply changing the HBA, the HBD, and/or the mole ratio in which they are combined, allows for the design of task-specific DESs^15,16^ with a myriad of applications, ranging from biocatalysis to electrochemistry.^5,17–21^

DES key attributes include remarkably low melting points, negligible vapor pressure, and high thermal stability, which enable their use across a wide range of processing temperatures.^19,22^ Based on this, DESs are potentially quite suitable as novel heat transfer fluids.^23^ Within this context, understanding their thermal properties is crucial for an efficient engineering design. In particular, thermal conductivity (*k*) and thermal diffusivity (α) dictate performance in heat exchange operations, providing insights about the DES ability to conduct heat in the former case and reflecting the rate of heat propagation relative to its capacity to store thermal energy in the latter one.

There are several works reporting on the thermal conductivities of different DESs. Neat DESs typically exhibit low to moderate thermal conductivities (i.e., within the range of 0.15–0.24 W m^–1^ K^–1^ at 25 °C) no matter the HBA and HBD used and/or the molar ratio in which they are combined.^24–27^ In this scenario, the addition of controlled amounts of water (with *k* well above those of DESs) may offer an effective strategy for tuning *k* and α. Interestingly, some DES aqueous dilutions have exhibited a certain deviation from ideality in *k*. It has been observed that the composition at which the deviation reaches a maximum and the magnitude of the deviation depends on the DES composition.^28^

With other different properties (e.g., density (ρ) and dynamic viscosity (η), among the most common),^29–34^ deviation from ideality has been observed in mixtures of DES and water due to the disruption of the original DES HB network to form new, and frequently stronger, water–DES interactions. This happens with the initial introduction of water molecules that, being highly polar and having a strong propensity for hydrogen bonding, do not merely dilute the mixture but actively compete with the existing interactions between the HBA and HBD components of the DES. Ultimately, these interactions determine not only the composition at which the deviation reaches a maximum but also the magnitude of the deviation itself. Whether the deviation from ideality in k follows the same pattern as that followed by other excess properties has not yet been investigated. It is worth noting that the study of mixtures of DES and water is of interest, given the myriad of applications where they can be used.^35^

Herein, we aim to investigate k and some other different excess properties of several aqueous solutions of a common DES, such as reline; this is the mixture of ChCl and U in a 1:2 mol ratio (e.g., ChCl:2U)^1^ and another less common DES such as that formed between methyltriphenylphosphonium bromide and Gly in a 1:3 mol ratio (e.g., M3PhPBr:3Gly).^36–38^ The determination of the heat capacities (C_p_) also allowed us to determine α. Moreover, we also used Brillouin spectroscopy to compare the hypersonic velocity (υ_H_) with regular ultrasound velocity (υ_U_) and to determine the excess adiabatic compressibility (Δβ_S_). The deviation from ideality of all these properties was compared with those obtained from the excess volume and excess viscosity.

## 2 Experimental Section

Choline Chloride (ChCl) was purchased from VWR. Urea (U) was purchased from Merck. Methyltriphenyl phosphoniumbromide (M3PhPBr) and glycerol (Gly) were purchased from Sigma-Aldrich. All of the reagents were used as received, except ChCl, which was dried at 90 °C overnight prior to its use (Table 1). After drying, the H_2_O content was negligible, as determined by Karl Fischer titration using a Titrando 888. Neat DESs were obtained by physical mixing of the individual components in the selected ratio and subsequent thermal treatment at 60 °C for 2 h to obtain a transparent liquid. Dilutions in H_2_O were obtained after the addition of the desired amount of deionized water. Deionized water was prepared in the lab with the ELGA VEOLIA system.

**Table 1:** Name and Abbreviations of the Chemical Compounds Used, with Their IUPAC Name, Source, and CAS Number, Their Initial Mole Fraction Purity, and the Water Content for ChCl and Gly

Name	Choline Chloride	Urea	Methyltriphenilphosphonium Bromide	Glycerol
**Abbreviation**	ChCl	U	M3PhPBr	Gly
**IUPAC Name**	2-Hydroxy-*N*,*N*,*N*-trimethylethan-1-aminium chloride	Carbonic diamide	Methyltri(phenyl)phosphanium bromide	Propane-1,2,3-triol
**Source**	VWR	Merck	Aldrich	Aldrich
**CAS Number**	67-48-1	57-13-6	1779-49-3	56-81-5
**Purity**	≥98.0%	≥98.0%	98%	≥99.5%
**Purification method**	drying	none	none	none
**Analysis method**	titration	none	none	titration
**Water Content (wt%)** ^a^	≤2.0			≤0.08
**Water Content** **(wt%)** ^b^	≤0.25			

a Before drying.

b After drying.

The density (ρ) and ultrasonic velocity (ν_U_) were measured at 25 °C and 0.1 MPa using the Anton Paar DMA5000 density meter. Automatic bubble detection inside the density meter ensured that no bubbles exist in the U tube measuring cell during the experiment. Before measurements, the measuring tube was cleaned with ultrapure water, rinsed with isopropyl alcohol, and then dried under air flow. The density of air (ρ = 1.2 kg m^–3^) was measured at the lowest end of the density spectrum. Other reference fluids used to calibrate the density meter were those provided by Anton Paar (e.g., dodecane, oil, water, glucose/water, and sodium bromide, with ρ = 750, 830, 998, 1040, and 1250 kg m^–3^, respectively). Measurements were repeated three times, and averaged values were reported. Standard uncertainties were *u*(T) = 0.05 °C and *u*(P) = 10 kPa, and expanded (k = 2) relative uncertainties were *U*_*r*_(ν_U_) = 0.0015 and *U*_*r*_(ρ) = 0.0005.

The measurements of dynamic viscosity (η) were carried out at 25 °C and 0.1 MPa using an Anton Paar Lovis 2000 ME viscosity meter, where the falling ball automated technique was applied. The viscosity was measured by combining the ball with a capillary of different diameters (1.59, 1.8, and 2.5 mm) and calibrating it with the standard liquids (N7.5, N26, and N100 viscosity oils). Measurements were repeated three times, and averaged values were reported. Standard uncertainties were *u*(T) = 0.05 °C and *u*(P) = 10 kPa, and the expanded (k = 2) relative uncertainty was *U*_*r*_(η) = 0.02.

^1^H NMR analyses of all samples were conducted using a Bruker Avance 500 spectrometer operating at 500 MHz with a broad-band fluorine observation (BBFO) probe capable of producing *z*-axis gradient pulses. The ^1^H NMR parameters employed included a 30° pulse, an acquisition time of 3.1719 s, a relaxation delay of 1 s, and a total of 16 to 32 scans. All samples were placed in capillary tubes and analyzed by using deuterated chloroform (CDCl_3_) as an external reference. The spectra of aqueous dilution of M3PhPBr:3Gly were acquired after setting the temperature to 50 °C using a Bruker Variable Temperature BVT 3000. The peaks were identified, and spectra were processed using the software MestReNova. Thermal conductivity (k) of all samples was analyzed using a Trident thermal analyzer (C-Therm Technologies Ltd., Canada), employing the Modified Transient Plane Source (MTPS) technique. The standard uncertainty was *u*(*k*) = 0.02 W m^–1^ K^–1^. In this method, a single-sided interfacial heat-reflectance sensor applies a momentary constant heat source to the sample. The applied current produces a rise in temperature at the interface between the sensor and the sample, which induces a change in the voltage drop across the sensor. The rate of increase in the sensor voltage is used to determine the thermal properties of the sample. Typically, the measurement pulse lasts between 1 and 3 s. In this case, since the samples were liquids, an accessory with an O-ring surrounding the sensor was used to prevent leakage. This O-ring assembly is placed directly onto the MTPS sensor to ensure proper containment of the liquid samples during measurement. Heat capacity (C_p_) measurements were performed by using a Discovery TA DSC instrument. Hermetically sealed Tzero aluminum pans were used as sample holders. Nitrogen was used as the purge gas at a flow rate of 50 mL min^–1^. The cell constant and temperature were previously calibrated using certified indium (156.60 °C; ΔH_f_ = 28.69 J g^–1^). The calibration curve for C_p_ measurements was obtained in triplicate by using a sapphire disk. C_p_ measurements were carried out between 10 and 35 °C at a heating rate of 5 °C min^–1^, with 10 min isothermal holds before and after the scan. All measurements were performed in triplicate, alternating each sample measurement with a sapphire run under identical conditions for verification. Thermograms are presented using the exotherm-up (Exo up) convention. Brillouin spectra were recorded using a Sandercock 3 + 3 Pass Tandem Fabry–Pérot interferometer as the Brillouin spectrometer,^39^ and the light source was a DPPS laser working at a wavelength (λ_0_) of 532 nm. In this case, the liquid samples were placed in optical cuvettes (Starna) with an optical path length of 1 mm. Experiments were performed using backscattering and 90° scattering geometry simultaneously. The simultaneous recording of both scattering geometries required the use of a neutral filter for the backscattering component, and we also had to reduce the intensity of the central peak. The Brillouin peaks were fitted by using a Lorentzian function with an adequate background function. The constraints associated with this experimental setup made it impossible to apply a typical damped harmonic oscillator model. The value of the compressibility of the real mixture (β_S_) was obtained directly from the Brillouin measurement of the hypersonic sound propagation velocity (ν_H_) and the ρ of the different aqueous dilutions upon the adoption of the Newton–Laplace equation:

1 $$\beta_{S}=\frac{1}{\rho \nu_{H}^{2}}$$

In the case of ideal mixing, when we do not account for any mixing volume, the adiabatic compressibility of the mixture can be written as

2 $$\beta_{Si}=\beta_{H_{2}O}+\left(\beta_{DES}-\beta_{H_{2}O}\right)X_{DES}$$

where β_DES_ is the compressibility of the neat DES (obtained experimentally from the measurement of ν_H_), $\beta_{H_{2}O}$ is the compressibility of H_2_O (obtained experimentally from the measurement of ν_H_ for water), and Χ_DES_ is the molar fraction of DES in the respective aqueous dilutions.

## 3 Results and Discussion

We started with the measurement of both the thermal conductivity (*k*) and the heat capacity (C_p_) for the determination of the thermal diffusivity (α), defined as the ratio of thermal conductivity to the volumetric heat capacity (α = k/ρC_p_, where ρ is the density) (Tables 1 and 2) For ChCl:2U, *k* was 0.255 W m^–1^ K^–1^ at 25 °C (Table 2), in agreement with previous data found by other authors (between 0.241 and 0.252 W m^–1^ K^–1^, Table S1).^24,28,40^ Meanwhile, for M3PhPBr:3Gly, *k* was 0.203 W m^–1^ K^–1^ at 25 °C (Table 3). This value was also within the range of those of other M3PhPBr-based DESs prepared with ethylene glycol or triethylene glycol (between 0.155 and 0.185 W m^–1^ K^–1^ at 25 °C, depending on the specific composition, Table S1).^41^ For ChCl:2U, *k* was higher than that found for M3PhPBr:3Gly. Actually, *k* for ChCl:2U was also higher than that for any of the other studied DESs (e.g., 0.206 W m^–1^ K^–1^ for ethaline, 0.198 W m^–1^ K^–1^ for maline, or 0.223 W m^–1^ K^–1^ for glyceline, among others).^27^ We also measured C_p_ for ChCl:2U and M3PhPBr:3Gly (Tables 2 and 3). For ChCl:2U, C_p_ was 2076 J kg^–1^ K^–1^ at 25 °C, in agreement with previous data found by other authors (ca. 2088 J kg^–1^ K^–1^, Table S1).^42^ For M3PhPBr:3Gly, C_p_ was 1705 J kg ^–1^ K^–1^ at 25 °C, also in agreement with previous data found by other authors (ca. 1658 J kg^–1^ K^–1^, Table S1).^43^ Finally, we measured ρ for ChCl:2U and M3PhPBr:3Gly (Tables 2 and 3). For ChCl:2U, ρ was 1200.1 kg m^–3^ at 25 °C, in agreement with previous data found by other authors (ca. 1196.6 kg m^–3^).^44^ For M3PhPBr:3Gly, ρ was 1296.7 kg m^–3^ at 25 °C, in agreement with previous data found by other authors (Table S1).^45–48^

**Table 2:** Thermal Conductivities, Heat Capacities, Densities, and Thermal Diffusivities of ChCl:2U and Aqueous Dilutions Thereof^a^

ChCl:2U content (wt%)	Thermal conductivity (*k*, W m^–1^ K^–1^)	Heat capacity (C_p_, J kg^–1^ K^–1^)	Density (ρ, kg m^–3^)	Thermal diffusivity (α, m^2^ s^–1^)
100	0.255	2076	1200.1	1.02 × 10^–7^
90	0.279	2265	1182.2	1.04 × 10^–7^
80	0.301	2405	1163.1	1.08 × 10^–7^
70	0.332	2606	1142.9	1.12 × 10^–7^
60	0.365	2868	1122.3	1.14 × 10^–7^
30	0.472	3522	1059.3	1.27 × 10^–7^
0	0.601	4186	997.9	1.44 × 10^–7^

a Data were obtained at 25 °C and at 0.1 MPa; standard uncertainty was *u*(*k*) = 0.02 W m^–1^ K^–1^ and expanded (*k* = 2) relative uncertainty was *U_r_* (ρ) = 0.0005.

It is worth noting that all these data were obtained considering the DESs as a mixture of two components rather than as a pseudocomponent. This definition determines the DES molecular weight (*M*_DES_). For instance, *M*_ChCl2U_ is 86.58 g mol^–1^ when considering ChCl:2U as a mixture of two components (i.e., *M*_ChCl:2U_ = *f*_ChCl_ *M*_ChCl_ + *f*_U_ *M*_U_, with *f*_ChCl_ = 1/3 and *f*_U_ = 2/3), whereas M*_ChCl:2U_ is 259.74 g mol^–1^ when considering ChCl:2U as a pseudocomponent (i.e., M*_ChCl:2U_ = *M*_ChCl_ + 2 *M*_U_). Moreover, *M*_M3PhPBr3Gly_ is 158.37 g mol^–1^ when considering M3PhPBr:3Gly as a mixture of two components (i.e., *M*_M3PhPBr3Gly_ = *f*_M3PhPBr_ *M*_M3PhPBr_ + *f*_Gly_ *M*_Gly_, with *f*_M3PhPBr_ = 1/4 and *f*_Gly_ = 3/4), whereas M*_M3PhPBr3Gly_ is 633.49 g mol^–1^ when considering M3PhPBr:3Gly as a pseudocomponent (i.e., M*_M3PhPBr3Gly_ = *M*_M3PhPBr_ + 3 *M*_Gly_).

Then, we measured k and C_p_ for aqueous dilutions of ChCl:2U and M3PhPBr:3Gly and determined their respective α values (Tables 2 and 3).^49^ It is worth noting the agreement between our measurements of C_p_ for aqueous dilutions of ChCl:2U with previous data found by other authors.^42,50^ Moreover, while neither C_p_ nor α values exhibited a significant deviation from ideality (data not shown),^51^ k indeed did it (excess thermal conductivity, Δk, in Figure 1a) with negative deviations that reached a minimum for DES contents within the 70–85 wt% range, depending on the treatment of the DESs as a mixture of two components or as a pseudocomponent (Table 4). The use of one or the other is by no means trivial for the determination of ideal data of DES dilutions, as, for the same weight percentage (wt%) of one of the components in the mixture, the mole fractions will differ (i.e., χ_1_ ≠ χ*_1_). Thus, our group and others have described how, in liquid binary mixtures of ChCl:2U with different solvents, this discrepancy between χ_1_ and χ*_1_ influences the absolute excess values, as well as the mole fraction at which the maximum deviation occurs (Figure 1).^44,52,53^

**Table 3:** Thermal Conductivities, Heat Capacities, Densities, and Thermal Diffusivities of M3PhPBr:3Gly and Aqueous Dilutions Thereof^a^

M3PhPBr:3Gly content (wt%)	Thermal conductivity (k, W m^–1^ K^–1^)	Heat capacity (C_p_, J kg^–1^ K^–1^)	Density (ρ, kg m^–3^)	Thermal diffusivity (α, m^2^ s^–1^)
100	0.203	1705	1296.7	0.92 × 10^–7^
97	0.215	1990	1286.9	0.84 × 10^–7^
93	0.227	1940	1273.9	0.92 × 10^–7^
90	0.234	2116	1263.9	0.88 × 10^–7^
85	0.252	2301	1247.2	0.88 × 10^–7^
80	0.268	2428	1230.3	0.90 × 10^–7^
70	0.305	2762	1197.1	0.92 × 10^–7^
50	0.386	3279	1133.4	1.04 × 10^–7^
20	0.511	4045	1047.4	1.21 × 10^–7^
0	0.601	4186	997.9	1.44 × 10^–7^

a Data were obtained at 25 °C and at 0.1 MPa; standard uncertainty was *u*(*k*) = 0.02 W m^–1^ K^–1^ and expanded (*k* = 2) relative uncertainty was *U_r_* (ρ) = 0.0005.

**Figure 1:**
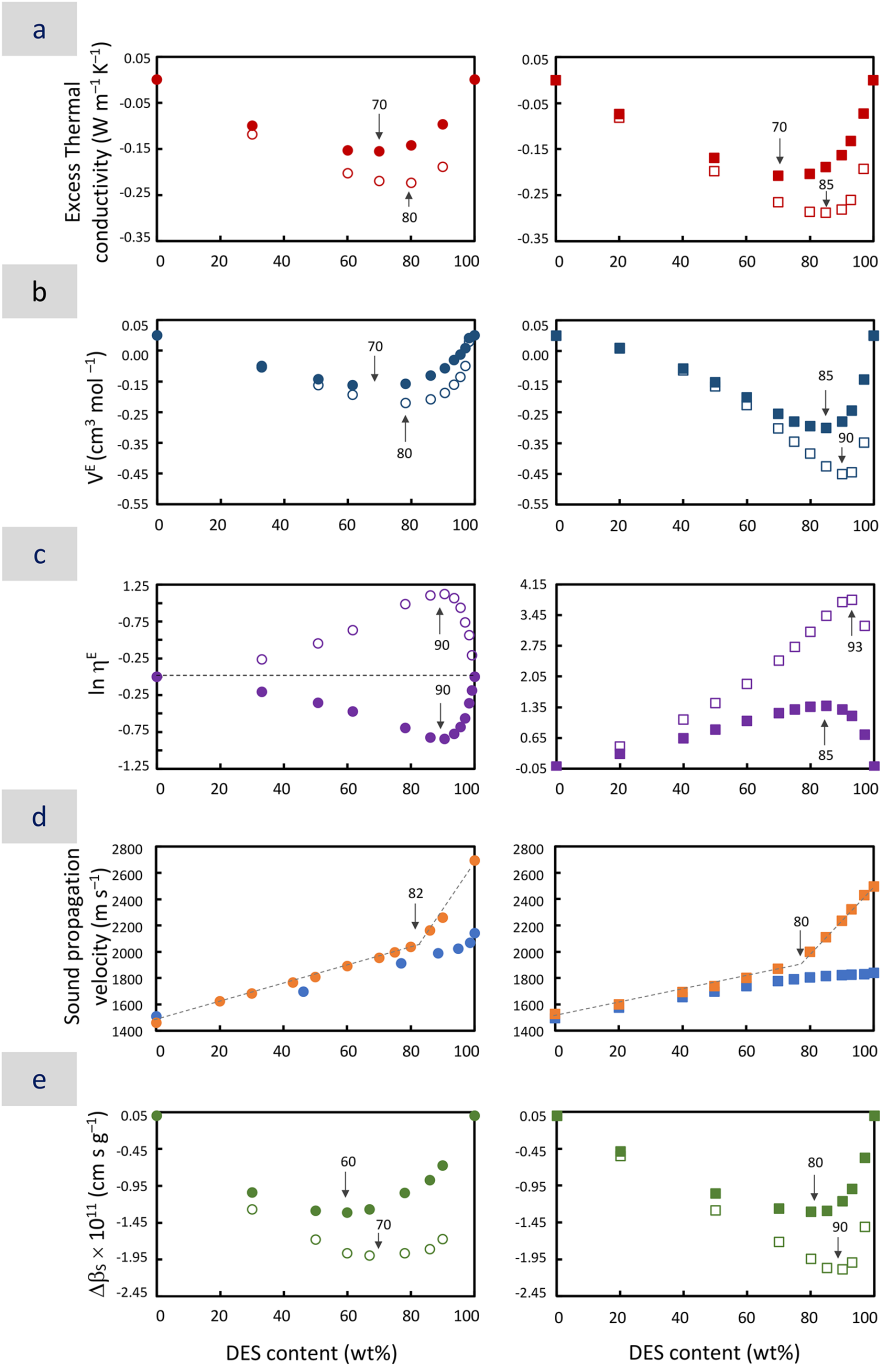
Plots of different excess properties obtained at 25 °C and 0.1 MPa versus DES content (in wt%) for aqueous dilutions of ChCl:2U (left column, circle symbols) and M3PhPBr:3Gly (right column, square symbols). Excess properties include (a) excess thermal conductivity (red symbols), (b) *V* ^E^ (blue symbols), (c) ln η^E^ (purple symbols), (d) hypersonic (orange symbols) and ultrasonic (light blue symbols) sound propagation, and (e) Δβ_S_ (green symbols). Solid or open symbols were used for DESs considered as a mixture of two components or as a pseudocomponent, respectively. The arrows point to the DES contents at which the deviations from ideality are maxima. Data can be found in Tables 1, 2 and S2, S3, S4, S5, S6, and S7. Standard uncertainties were *u*(T) = 0.05 °C, *u*(P) = 10 kPa, and *u*(*k*) = 0.02 W m^–1^ K^–1^, and expanded (k = 2) relative uncertainties were *U_r_* (ν_U_) = 0.0015, *U_r_* (ρ) = 0.0005, and *U_r_* (η) = 0.02.

**Table 4:** Specific DES Contents for which the ChCl:2U and M3PhPBr:3Gly Aqueous Dilutions Deviated More from Ideality for Different Excess Properties, Depending on Their Treatment as Mixtures of Two Components or as a Pseudo-Component (*)^a^

		**DES content (wt%)**			
		ChCl:2U	ChCl:2U*	M3PhPBr:3Gly	M3PhPBr:3Gly*
**Deviation Excess Property**	Δ*k*	70	80	70	85
	*V* ^E^	70	80	85	90
	ln η^E^	90	90	85	93
	Δβ_S_	60	70	80	90
					
**Deviation of ν**_**H**_ **from ν**_**US**_		82		80	
**Transition from noncoalescent signals to coalescent ones**		75		75	

a The table also includes the specific DES contents for (**) deviation of υ_H_ from υ_U_ and for (***) transition from non-coalescent signals to coalescent ones occurred in the ^1^H spectra. Note that these deviations are not affected by the treatment of DESs as two components or as a pseudo-component.

For the purpose of comparing the deviation from ideality in Δk to deviations of other properties, excess volume (*V* ^E^) and excess viscosity (Δη) were also studied. *V* ^E^ for a liquid binary mixture is calculated from the density of the mixture (ρ) and the densities (ρ_1_ and ρ_2_), the mole fractions (χ_1_ and χ_2_), and the molecular weights (*M*_1_ and *M*_2_) of the individual components using the following equation (eq 3):

3 $$V^{E}=\chi_{1}M_{1}/\rho +\chi_{2}M_{2}/\rho -\chi_{1}M_{1}/\rho_{1}-\chi_{2}M_{2}/\rho_{2}$$

Meanwhile, Δη is calculated from the viscosity of the mixture (η) and the viscosities (η_1_ and η_2_) and the mole fractions (χ_1_ and χ_2_) of the individual components using the following equation (eq 4):

4 $$\Delta \eta =\eta -\chi_{1}\eta_{1}-\chi_{2}\eta_{2}$$

However, it is more common to calculate the excess logarithmic viscosity (ln η^E^) using the following equation (eq 5):

5 $$\ln\eta^{E}=\ln\eta -\chi_{1}\ln\eta_{1}-\chi_{2}\ln\eta_{2}$$

given the nonlinear relationship between viscosity and molar fraction.^54^

For aqueous dilutions of ChCl:2Urea, *V* ^E^ and ln η^E^ data have been obtained in different works,^44,52,55–60^ so we decided to use the data of one of them (Table S2)^52^ based on a previous analysis we did with many of them to determine the most reliable data.^53^ For aqueous dilutions of M3PhPBr:3Gly, we performed our own measurements to obtain the data (Table S3). In any case, density and viscosity results for M3PhPBr:3Gly were consistent with previously reported data.^46^ *V* ^E^ is the difference between the actual volume of a mixture and the volume predicted by simply adding the volumes of the pure components (ideal mixing). Packing efficiency and intermolecular force changes are responsible for positive or negative deviations. For instance, as for other aqueous solutions of DESs,^22–27^ ChCl:2U and M3PhPBr:3Gly also displayed negative deviations that reached a minimum for certain DESs (Figure 1b, Table 4). Negative deviations have been ascribed to the occurrence of stronger HB interactions between H_2_O and the DES components than between those formed separately by the DES components and H_2_O molecules. Meanwhile, the most prominent sensitivity of ln η^E^ was due to the presence of specific molecular aggregates or ordered structures in which the strong molecular associations make the mixture much harder to flow, leading to positive deviations as those observed for M3PhPBr:3Gly (Figure 1c). This is the case for most aqueous dilutions of DESs,^29–34^ except for ChCl:2U in which, when considered a mixture of two components, the deviation was negative (Figure 1c, Table 4)^44,55–60^ as for mixtures with negligible (or, at least, neither strong nor numerous) HB interactions between components.^61–63^ This anomalous behavior has been discussed in detail in one of our previous works,^53^ and it will not be further discussed here.

With the aim of understanding the results described above, we performed DES characterization by FTIR and ^1^H NMR spectroscopies. The ATR spectra of ChCl:2U and aqueous dilutions thereof are depicted in Figure 2a. The spectrum of pure ChCl:2U was quite similar to that reported in previous works.^64^ Among the most significant bands, we could mention the broad band centered at ca. 3250 cm^–1^ and the two vibrational ones at ca. 1600 and 1660 cm^–1^. The former one at ca. 3250 cm^–1^ was composed of (1) the N–H asymmetric and symmetric stretches of U at ca. 3400 and 3310 cm^–1^, respectively, (2) the overtone modes of C=O stretching and −NH_2_ bending modes involved in a Fermi resonance with the N–H vibrations of U at ca. 3185 cm^–1^), and (3) the O–H stretching vibration of the HB formed between Ch^+^ and Cl^–^ at ca. 3255 cm^–1^.^65^ The latter ones at ca. 1600 and 1660 cm^–1^ corresponded to the mixed modes of −NH_2_ bending and C=O stretching vibrations of U.^66,67^ Finally, the weak peaks observed at 3000 cm^–1^ were assigned to CH stretching modes of the aliphatic groups of Ch^+^ (e.g., −CH_3_ and −CH_2_−). As described elsewhere, increasing the hydration resulted in a loss of spectral structure due to a broadening of the infrared band centered at about 3250 cm^–1^ due to the increasing contribution of the O–H stretch vibrations of water to the signal. Meanwhile, the bands at ca. 1600 and 1660 cm^–1^ decreased in amplitude along with an increase in water content. Actually, they basically disappeared at water contents of ca. 60 wt%, while some new peaks appeared, one strong at ca. 1640 cm^–1^ assigned to the bending mode of H_2_O and one weak at ca. 1705 cm^–1^ assigned to N–H vibrations of U not involved in HBs with Cl^–^.^68^ The FTIR spectra of M3PhPBr:3Gly and the aqueous dilutions thereof are also depicted in Figure 2b. In this case, we observed the O–H stretching vibration of Gly (broader and red-shifted as compared to that of bare Gly due to the formation of HBs) at ca. 3300 cm^–1^, the CH stretching modes of the aliphatic groups of Gly (−CH_2_−), as well as the aliphatic and aromatic groups of M3PhPBr at ca. 2880–2935 cm^–1^, the strong skeletal vibration of —C=C— of M3PhPBr at ca. 1435 cm^–1^, and the C–O streching and the P–C vibrations overlapping at ca. 1000–1100 cm^–1^.^69^ Interestingly, the increase in hydration resulted in just a gradual decrease in the intensity of the bands assigned to M3PhPBr and Gly and a gradual increase in the band at ca. 1640 cm^–1^ assigned to H_2_O.

**Figure 2:**
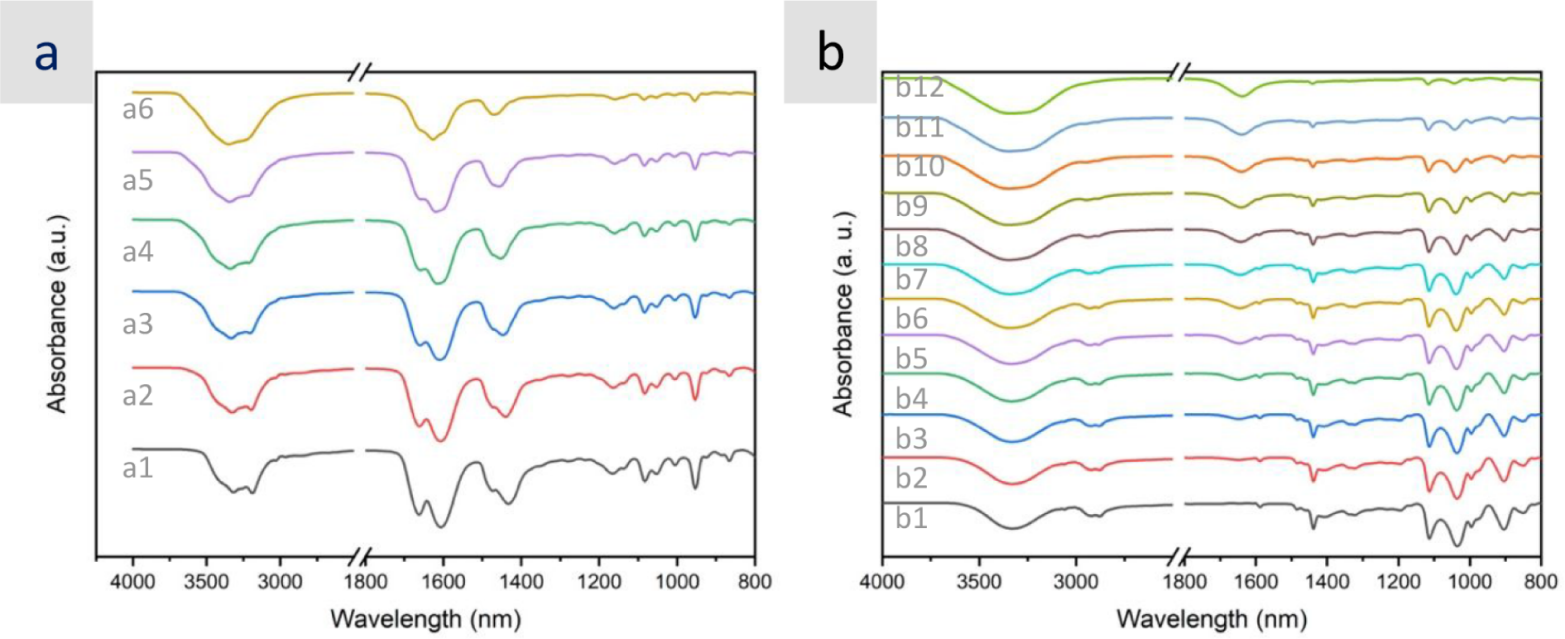
ATR spectra of aqueous dilutions of (a) ChCl:2U with DES contents of (a1) 100, (a2) 90, (a3) 80, (a4) 70, (a5) 60, and (a6) 30 and (b) M3PhPBr:3Gly with DES contents of (b1) 100, (b2) 97, (b3) 93, (b4) 90, (b5) 85, (b6) 80, (b7) 75, (b8) 70, (b9) 60, (b10) 50, (b11) 40, and (b12) 20 wt%.

The effects that H_2_O addition caused on the different DESs were also investigated by ^1^H NMR spectroscopy (Figure 3 and Figure S1). The ^1^H NMR spectra of ChCl:2U and aqueous dilution thereof have been discussed in detail elsewhere.^70^ In this case, the most remarkable results were the coalescence of the exchangeable protons at a certain H_2_O content (OH of ChCl and H_2_O; see Figure S1) and, with the help of results obtained from Brillouin spectroscopy, the identification of this transition of the exchangeable proton signals with the transition from an extensive HB network (i.e., the so-called water-in-DES regime) to a simple solution of the individual DES components (i.e., the so-called DES-in-water regime) along with the increase of H_2_O content.^70,71^ The ^1^H NMR spectrum of M3PhPBr:3Gly displayed peaks assigned to nonexchangeable and exchangeable protons (Figure 3, Table S8). Among the nonexchangeable ones, we observed the phenyl and methyl groups of M3PhPBr (aromatic = CH– at ca. 7.33–7.07 ppm and CH_3_ at ca. 3.65 ppm, respectively) and the methyne and methylene groups of Gly (−CH– at ca. 3.23 ppm and −CH_2_– at 3.14–2.92 ppm, respectively) (Table S8). Meanwhile, among the exchangeable groups, we observed the −OH groups of Gly as two separate signals at ca. 4.25 and 4.15 ppm (Table S8). We could also observe a low intensity signal (with an integral of 0.3) at ca. 3.7 ppm, revealing the presence of small traces of H_2_O (Table S8). Based on this, the nondiluted sample should be considered as a sample with an H_2_O content of 0.4 wt%. Further addition of H_2_O, to obtain aqueous M3PhPBr:3Gly dilutions, resulted in downfield shifts in every signal because of the formation of HBs (Figure 3, Table S8). Particularly interesting was the evolution of the signals assigned to exchangeable protons. In this regard, the addition of H_2_O resulted in not only the downfield shift of every exchangeable proton but also their progressive coalescence from three separate signals to two in a first place (i.e., by the coalescence of those of the OH groups of Gly at water contents of 85 wt%) and just one afterward (i.e., by the coalescence of this one with that of H_2_O at water contents of ca. 75 wt%). Down-field shifts were indicative of the participation of H_2_O in the original HB complex structure formed between DES components, thus creating a more extensive HB network than that originally formed between DES components. Meanwhile, coalescent signals are typical of diluted solutions where solutes are fully solvated by H_2_O molecules, and proton became more mobile than when “locked” in stable, long-lived HB clusters characteristic of concentrated mixtures.^72–74^ Interestingly, this transition of the exchangeable proton signals, from separated to coalescent, agreed with the behavior described above for aqueous ChCl:2U dilutions.

**Figure 3:**
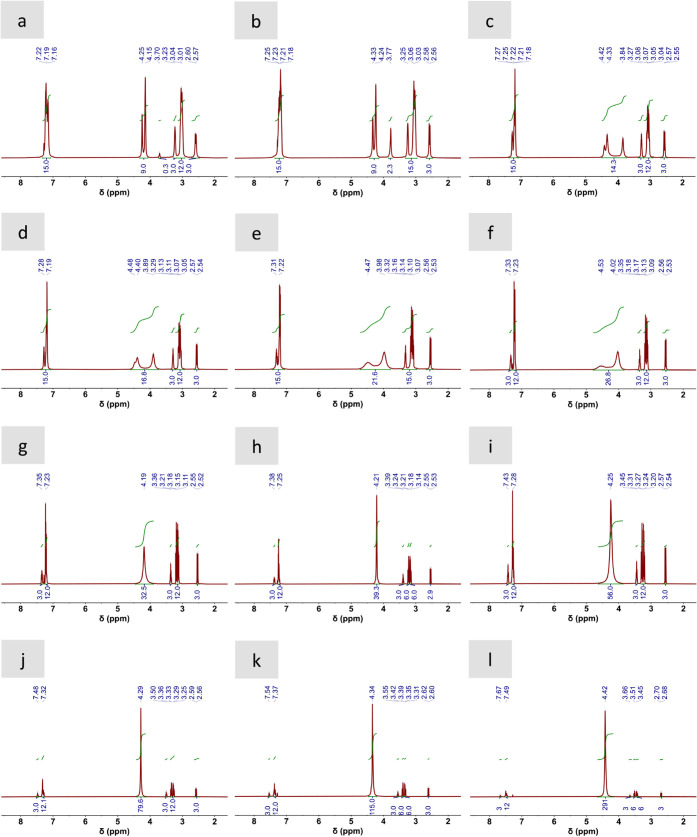
^1^H NMR spectra of aqueous dilutions of M3PhPBr:3Gly with DES contents of (a) 100, (b) 97, (c) 93, (d) 90, (e) 85, (f) 80, (g) 75, (h) 70, (i) 60, (j) 50, (k) 40, and (l) 20 wt%. Data can be found in Table S8.

Thus, our next step was studying both DESs and their respective aqueous dilutions by Brillouin spectroscopy (Figure 1d, Tables S4 and S5). It is worth noting that Brillouin spectroscopy is particularly suitable for detecting the local structural rearrangements in liquid mixtures of HB cosolvents (MeOH, EtOH, t-BuOH, etc.), polymers (PEG), and even hydrated salts with either water or organic solvents.^75–80^ In all these mixtures, the plot of hypersonic velocity (ν_H_, obtained from Brillouin measurements) versus one of the components’ content exhibits a deviation from the ideal behavior (i.e., the velocity obtained by ultrasonic experiments, ν_U_).^81^ It is worth noting that our measurements of ν_U_ for neat H_2_O and ChCl:2U (e.g., 1507 and 2140 m s^–1^, respectively) were in agreement with previous data found by other authors.^42,82^ Thus, while the measurement of ν_H_ and ν_U_ for neat H_2_O provides basically the same values (ca. 1460 and 1507 m s^–1^, respectively, in agreement with previous works reporting ν_H_ and ν_U_ for neat H_2_O),^75–80^ ν_H_ and ν_U_ typically differ when the DES content surpasses a certain value.^70,71^ These discrepancies have been ascribed to a relaxation phenomenon occurring with the first formation of a continuous structure of DES domains, when transitioning from the DES-in-water regime (comprising individually solvated DES components to isolated DES domains in a continuous structure of H_2_O) to the water-in-DES one (comprising a cocontinuous structure of H_2_O and DES domains to isolated H_2_O domains in a continuous structure of DES). As for the^1^H NMR results, the behavior exhibited by ChCl:2U and M3PhPBr:3Gly dilutions was quite similar, with ν_H_ deviating from ν_U_ at certain DES contents (Figure 1d, Table 4). Interestingly, the behavior observed in both systems bore a resemblance to that observed for PEG/H_2_O binary mixtures.^78^

We could also analyze the concentration dependence of Δβ_S_ by the difference between the real compressibility of the mixture and the ideal one (Tables S6 and S7).^83^ Δβ_S_ acts as a detailed probe into the internal “stiffness” or “compactness” of the liquid mixture at a molecular level.^84^ Its value is a direct consequence of the interplay between the strength of the intermolecular forces and the efficiency with which the different molecular shapes can pack together. Thus, the negative values of Δβ_S_ found over the whole compositional range were indicative of the building up of a tight local structure—more so than any built by the cosolvents individually—upon the formation of an extended HB network in which both DES and neat H_2_O were involved, reaching a maximum in rigidity at the DES contents where the deviation from ideality was maximum (Figure 1e, Table 4).

## 4 Conclusions

The present study allowed for the systematic analysis of the thermodynamic behavior of aqueous dilutions of ChCl:2U and M3PhPBr:3Gly, focusing first on the measurement of k, α, and C_p_ and then on the determination of a number of excess properties, including Δk, *V* ^E^, ln η^E^, and Δβ_S_, at 25 °C and ambient pressure. By comparing the deviations from ideality, this study confirmed the nonideal behavior of a wide range of properties of different nature and a correlation in the sign of the deviation of both DESs (except for ln η^E^ for ChCl:2U:H_2_O mixture that has been previously discussed) as a consequence of the common molecular origin for these thermodynamic deviations. We have also compared ν_H_ versus ν_U_, for which we found higher ν_H_ than ν_U_ in the mixtures when the DES contents reached a threshold content that revealed the occurrence of HB breaking/forming between H_2_O and the DES components. Moreover, the transition from nonideality to ideality can also be correlated to the transition from noncoalescent to coalescent signals in the ^1^H NMR spectra with protons “locked” in stable, long-lived HB clusters in nonideal mixtures that became more mobile when clusters were broken upon dilution. Thus, the critical finding of this study was that the specific composition at which the maximum deviation from ideality is reached for all measured excess properties was basically coincident among them as well as with that observed for the deviation between ν_H_ and ν_U_ and that observed for the transition from noncoalescent to coalescent ^1^H NMR signals (Table 4). This coincidence at the points of maximum deviation was somehow expected, considering that these measurements are all sensitive to the same aspects of the mixture microstructure (e.g., molecular interactions, packing efficiency, and structural changes). Interestingly, this trend was more universal for the M3PhPBr:3Gly aqueous dilutions than for the ChCl:2U ones, thus confirming the peculiarities already observed for the latter ones (for instance, the above-mentioned positive deviation experienced by ln η^E^).

## Supplementary Material



## Notes

Values of the density, viscosity, ultrasonic and hypersonic velocity, adiabatic compressibility, ^1^H NMR chemical shifts, and ^1^H NMR spectra (PDF)
