# Supplementary material for: Excess Properties of Aqueous Dilutions of Ammonium- and Phosphonium-Based Deep Eutectic Solvents
Source: J Chem Eng Data. 2026 Apr 28;71(5):2118–28. doi: 10.1021/acs.jced.6c00067 (PMC13186101; doi:10.1021/acs.jced.6c00067)
Supplement: Supplementary file 1 [file je6c00067_si_001.pdf]

Supporting Information

Excess properties of aqueous dilutions of ammonium- and phosphonium-based deep eutectic solvents

Xusheng Zhou,[‡] Wenjia Zhang,[‡] Manuel Cerro Carracedo,[‡] Rafael J. Jiménez-Riobóo, M. Luisa Ferrer, María C. Gutiérrez,* and Francisco del Monte*

Instituto de Ciencia de Materiales de Madrid-ICMM, Consejo Superior de Investigaciones Científicas-CSIC. Campus de Cantoblanco, 28049-Madrid (Spain),

[‡] These authors contributed equally to this work

* Corresponding authors: delmonte@icmm.csic.es; mcgutierrez@icmm.csic.es

ORCID number for F. del Monte: 0000-0002-2868-0023
ORCID number for M. C. Gutiérrez: 0000-0001-8612-7974

Table S1: Thermal conductivities, heat capacities and densities of ChCl:2U, P1Ph3Br:3Gly and P1Ph3Br-based DESs with ethylene glycol (EG) and triethylene glycol (TEG) found in this work and in previous works. Data was obtained at 25 °C and at 0.1 MPa. Standard uncertainty was $u(k)$ = 0.02 W m^{-1} K^{-1} and expanded (coverage factor = 2) relative uncertainty was $U_r(\rho)$ = 0.0005.

Type of DES	Thermal conductivity (k, W m^{-1} K^{-1})	Heat capacity (C_p, J kg^{-1} K^{-1})	Density (ρ, kg m^{-3})	Reference
ChCl:2U	0.255	2076	1200.1	This work
ChCl:2U	0.245	--	--	24
ChCl:2U	0.241	--	--	28
ChCl:2U	0.2522	--	1205.1	40
ChCl:2U	--	2088	1199.5	42
P1Ph3Br:3Gly	0.203	1705	1296.7	This work
P1Ph3Br:3EG	0.185	--	--	41
P1Ph3Br:3TEG	0.155	--	--	41
P1Ph3Br:3Gly	--	1658	--	43
P1Ph3Br:3Gly	--	--	1306.4	45
P1Ph3Br:3Gly	--	--	1296.5	46
P1Ph3Br:3Gly	--	--	1295.8	47
P1Ph3Br:3Gly	--	--	1307.1	48

Table S2: Density (ρ) and viscosity (*η*) of ChCl:2U and aqueous dilutions thereof. From reference 52. Data was obtained at 25 °C and at 0.1 MPa. Expanded (coverage factor = 2) relative uncertainties were U_r(ρ) = 0.0005 and U_r(η) = 0.02.

DES content (wt%)	Density (kg m^{-3})	Viscosity (Pa s)
100	1196.80	1.3980
98.2	1192.77	0.8901
97.1	1190.55	0.5474
95.5	1187.30	0.3115
93.5	1183.02	0.1820
91.0	1176.82	0.1017
86.1	1167.48	0.0516
78.2	1151.19	0.0246
61.6	1116.99	0.0104
50.7	1094.96	0.0035
33.0	1059.68	0.0023
0	997.9	0.0009

Table S3: Density (ρ) and viscosity (*η*) of M3PhPBr:3Gly and aqueous dilutions thereof. Data was obtained at 25 °C and at 0.1 MPa. Expanded (coverage factor = 2) relative uncertainties were U_r(ρ) = 0.0005 and U_r(η) = 0.02.

DES content (wt%)	Density (kg m^{-3})	Viscosity (Pa s)
100	1296.69	2.9260
97	1286.94	1.0650
93	1273.86	0.3677
90	1263.89	0.1972
85	1247.19	0.0852
80	1230.32	0.0470
75	1213.74	0.0257
70	1197.10	0.0165
60	1164.62	0.0082
50	1133.37	0.0047
40	1103.34	0.0030
20	1047.43	0.0015
0	997.9	0.0009

Table S4: Hipersonic (ν_H) and ultrasonic (ν_U) velocities of ChCl:2U and aqueous dilutions thereof. From reference 50. Data was obtained at 25 °C and at 0.1 MPa. Expanded (coverage factor = 2) relative uncertainty was $U_r(\nu_U) = 0.0015$.

DES content (wt%)	ν_H (m s^{-1})
100	2691.9
90	2256.4
86	2159.2
80	2035.7
75	1992.4
70	1952.5
60	1889.1
50	1807.6
43	1764.3
30	1681.5
20	1621.8
0	1460.0

DES content (wt%)	ν_U (m s^{-1})
100	2140
98.6	2067
94.8	2023
88.6	1988
76.9	1912
46.3	1696
0	1507

Table S5: Hipersonic (ν_H) and ultrasonic (ν_U) velocities of M3PhPBr:3Gly and aqueous dilutions thereof. Data was obtained at 25 °C and at 0.1 MPa. Expanded (coverage factor = 2) relative uncertainty was $U_r(\nu_U) = 0.0015$.

DES content (wt%)	ν_H (m s^{-1})	ν_U (m s^{-1})
100	2496.9	1841,82
97.0	2431.7	1830,94
93.0	2321.6	1825,08
90.0	2237.0	1822,27
85.0	2112.2	1815,76
80.0	2001.6	1805,68
75.0		1792,27
70.0	1871.2	1776,32
60.0	1802.2	1738,89
50.0	1739.3	1698,23
40.0	1693.2	1656,58
20.0	1602.4	1575,15
0	1460.0	1498

Table S6: Adiabatic compressibility ($\beta_S = 1/\rho v_H^2$) of ChCl:2U and aqueous dilutions thereof. Densities were obtained at 25 °C and at 0.1 MPa. Expanded (coverage factor = 2) relative uncertainty was $U_r(\rho) = 0.0005$.

DES content (wt%)	Density (kg m^{-3})	β_S (Pa^{-1})
100	1199.29	1.15×10^{-10}
90	1179.51	1.67×10^{-10}
86	1170.15	1.83×10^{-10}
78	1153.83	2.14×10^{-10}
67	1130.70	2.37×10^{-10}
60	1119.54	2.50×10^{-10}
50	1097.39	2.79×10^{-10}
30	1061.81	3.33×10^{-10}
0	997.9	4.70×10^{-10}

Table S7: Adiabatic compressibility (β_S) of M3PhPBr:3Gly and aqueous dilutions thereof. Data was obtained at 25 °C and at 0.1 MPa. Expanded (coverage factor = 2) relative uncertainty was $U_r(\rho) = 0.0005$.

DES content (wt%)	Density (kg m^{-3})	β_S (Pa^{-1})
100	1296.69	1.24×10^{-10}
97.0	1286.94	1.31×10^{-10}
93.0	1273.86	1.46×10^{-10}
90.0	1263.89	1.58×10^{-10}
85.0	1247.19	1.80×10^{-10}
80.0	1230.32	2.03×10^{-10}
70.0	1197.10	2.39×10^{-10}
50.0	1133.37	2.92×10^{-10}
20.0	1047.43	3.72×10^{-10}
0	997.9	4.70×10^{-10}

Table S8: ^{1}H NMR chemical shifts of aqueous dilutions of P1Ph3Br:3Gly with different DES contents.

DES content (wt%)	Chemical shifts (ppm) and number of H from integrals (nH)							
	**M3PhPBr**			**Glycerol**				**H_2O**
	Aromatic CH		CH_3	OH		CH	CH_2	
100 (99.6)	7.33-7.07		2.60-2.57	4.25	4.15	3.23	3.14-2.92	3.7
	15H		3H	9H (4.32-4.06)		3H	12H	0.3H
97	7.25	7.18	2.58-2.56	4.33	4.24	3.25	3.19-2.95	3.77
	15H (7.36-7.09)		3H	9H (4.43-4.14)		3H	12H	2.3H
93	7.27	7.18	2.57-2.55	4.42	4.33	3.27	3.20-2.91	3.84 (4.50-3.74)
	3H	12H	3H	9H+5H (4.50-3.74)		3H	12H	5H+9H
90	7.28	7.19	2.57-2.54	4.48	4.40	3.29	3.22-2.94	3.89 (4.61-3.73)
	3H	12H	3H	9H+7H (4.61-3.73)		3H	12H	7H+9H
85	7.31	7.22	2.56-2.53	4.47 (4.72-3.79)		3.32	3.28-2.97	3.98 (4.72-3.79)
	3H	12H	3H	9H+12H		3H	12H	12H+9H
80	7.33	7.23	2.56-2.53	4.53 (4.79-3.83)		3.35	3.23-3.04	4.02 (4.79-3.83)
	3H	12H	3H	9H+16H		3H	12H	16H+9H
75	7.35	7.23	2.55-2.52	4.19		3.36	3.21-3.11	4.19
	3H	12H	3H	9H+23.5H		3H	12H	9H+23.5H
70	7.38	7.25	2.55-2.53	4.21		3.39	3.24-3.14	4.21
	3H	12H	3H	9H+30H		3H	12H	9H+30H
60	7.43	7.28	2.57-2.54	4.25		3.45	3.31-3.20	4.25
	3H	12H	3H	9H+47H		3H	12H	9H+47H
50	7.48	7.32	2.59-2.56	4.29		3.50	3.36-3.25	4.29
	3H	12H	3H	9H+47H		3H	12H	9H+47H
40	7.54	7.37	2.62-2.60	4.34		3.55	3.42-3.31	4.34
	3H	12H	3H	9H+106H		3H	12H	9H+47H
20	7.67	7.49	2.70-2.68	4.42		3.66	3.55-3.38	4.42
	3H	12H	3H	9H+282H		3H	12H	282H+9H

Figure S1: ^{1}H NMR spectra of aqueous dilutions of ChCl:2U with DES contents (in wt%) of 100 (a), 98 (b), 96 (c), 90 (d), 86 (e), 80 (f), 75 (g), 70 (h), 60 (i), 50 (j), 43 (k), and 30 (l). Insets in (b), (c), (d), (e), (f), and (g) are included for better visualization of those exchangeable protons giving low intensity peaks. Reprinted from reference 50.

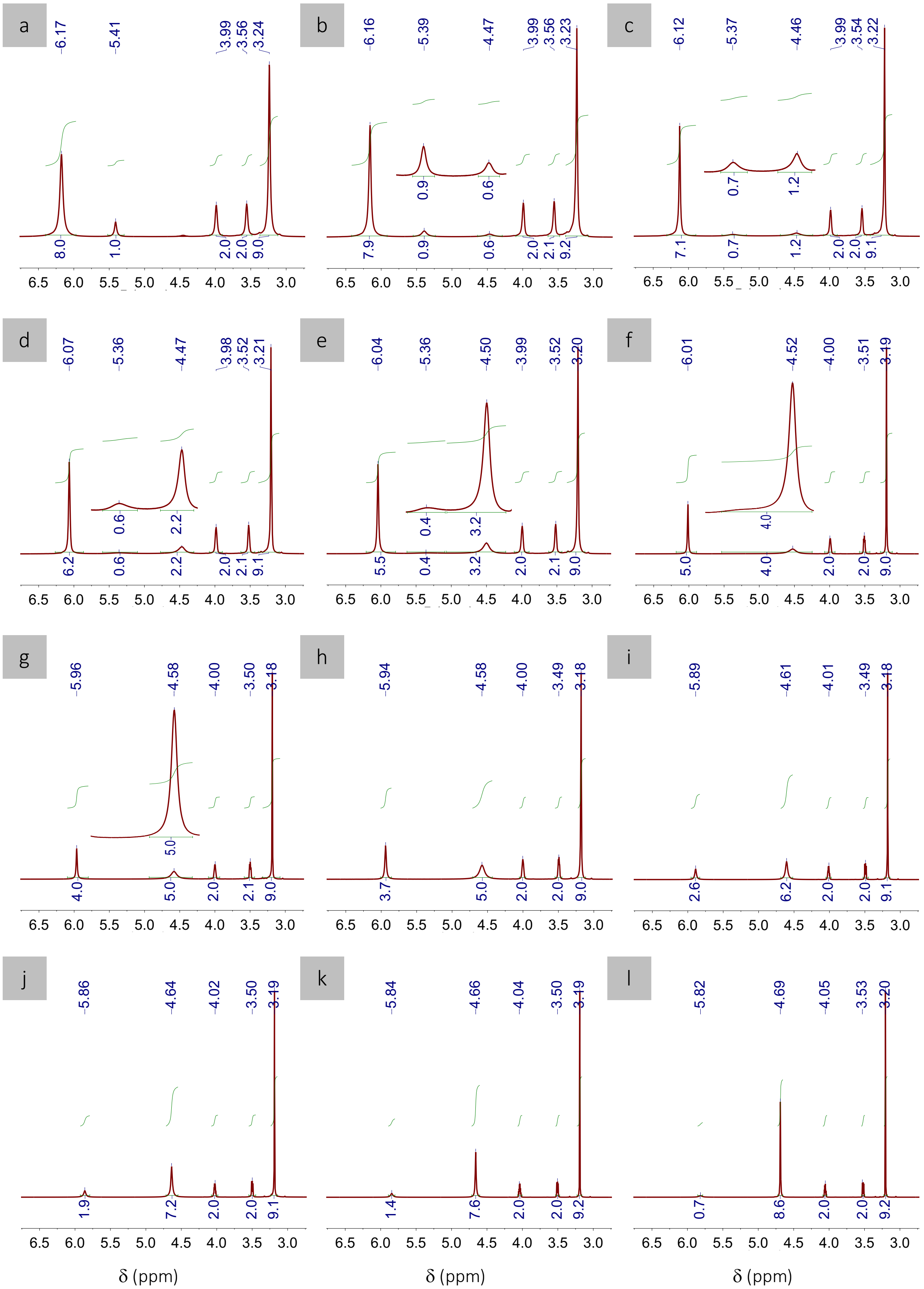
a
-6.17
-5.41
3.99
3.56
3.24
8.0
1.0
2.0
2.0
9.0
b
-6.16
-5.39
-4.47
3.99
3.56
3.23
0.9
0.6
7.9
0.9
0.6
2.0
2.1
9.2
c
-6.12
-5.37
-4.46
3.99
3.54
3.22
0.7
1.2
7.1
0.7
1.2
2.0
2.0
9.1
d
-6.07
-5.36
-4.47
3.98
3.52
3.21
0.6
2.2
6.2
0.6
2.2
2.0
2.1
9.1
e
-6.04
-5.36
-4.50
-3.99
-3.52
3.20
0.4
3.2
5.5
0.4
3.2
2.0
2.1
9.0
f
-6.01
-4.52
-4.00
-3.51
3.19
4.0
5.0
4.0
2.0
2.0
9.0
g
-5.96
-4.58
-4.00
-3.50
3.18
5.0
4.0
5.0
2.0
2.1
9.0
h
-5.94
-4.58
-4.00
-3.49
3.18
3.7
5.0
2.0
2.0
9.0
i
-5.89
-4.61
-4.01
-3.49
3.18
2.6
6.2
2.0
2.0
9.1
j
-5.86
-4.64
-4.02
-3.50
3.19
1.9
7.2
2.0
2.0
9.1
k
-5.84
-4.66
-4.04
-3.50
3.19
1.4
7.6
2.0
2.0
9.2
l
-5.82
-4.69
-4.05
-3.53
3.20
0.7
8.6
2.0
2.0
9.2
6.5 6.0 5.5 5.0 4.5 4.0 3.5 3.0
δ (ppm)
δ (ppm)
δ (ppm)
